# Glycation of vitronectin inhibits VEGF-induced angiogenesis by uncoupling VEGF receptor-2–*α*v*β*3 integrin cross-talk

**DOI:** 10.1038/cddis.2015.174

**Published:** 2015-06-25

**Authors:** L Wang, X Zhang, N Pang, L Xiao, Y Li, N Chen, M Ren, X Deng, J Wu

**Affiliations:** 1 Drug Discovery Research Center, Sichuan Medical University, Luzhou, Sichuan, People's Republic of China; 2 Division of Cardiovascular Medicine, Department of Medicine, University of Missouri School of Medicine, Columbia, MO, USA

## Abstract

Glycation of vessel wall proteins is thought to have an important role in the pathogenesis of vascular complications in diabetes mellitus. However, no previous study has implicated glycated vitronectin (VN) in the control of vascular endothelial growth factor (VEGF) signaling. To explore whether the glycation of VN affects angiogenic signaling and to understand the molecular mechanisms involved, we synthesized glycated VN by incubating VN with methylglyoxal (MGO) *in vitro* and identified the formation of glycated VN by an LC–ESI–MS/MS-based method. We tested the hypothesis that glycation of VN downregulates VEGF receptor-2 (VEGFR-2) activation by uncoupling the interaction between VEGFR-2 and *α*v*β*3. Unmodified and MGO-glycated VN were used as substrates for human umbilical vein endothelial cells (HUVECs). The effects of glycated VN on VEGF signaling in HUVECs were investigated. The glycation of VN inhibited VEGF-induced phosphorylation of VEGFR-2 and the intracellular signaling pathway downstream of VEGFR-2. Glycated VN inhibited the binding of VEGFR-2 to *β*3 integrin and inhibited the phosphorylation of *β*3 integrin. Furthermore, glycation of VN significantly decreased VEGF-induced migration of HUVECs *in vitro* and vessel outgrowth in an *ex vivo* angiogenesis model. Collectively, these data indicate that the glycation of VN inhibits VEGF-induced VEGFR-2 activation by uncoupling VEGFR-2–*α*v*β*3 integrin cross-talk. The glycation of VN causes a reduction in the migration of endothelial cells and vessel outgrowth. This may provide a mechanism for the failure of collateral sprouting in diabetic microangiopathy.

Type 2 diabetes is a chronic hyperglycemic condition that causes both microvascular and macrovascular complications. Impaired angiogenesis contributes to the development of various vascular complications in diabetes mellitus, and a number of abnormalities associated with angiogenesis have been observed in people with type 2 diabetes.^1, 2^ The accumulation of advanced glycation end products (AGEs) may have an important role in the neovasculature of vascular complications in diabetes.^3, 4, 5, 6^ Angiogenesis is triggered by angiogenic growth factors and vascular endothelial growth factor (VEGF) is a major angiogenic mediator under physiological and pathophysiological conditions.^7^ However, people with diabetes mellitus often show a poor response to therapeutic angiogenesis^8^ and develop VEGF resistance, an impairment in VEGF-induced signal transduction, which has been demonstrated as a molecular basis for the impaired angiogenesis in diabetes mellitus.^9^ The molecular mechanisms underlying VEGF resistance in diabetes mellitus are not fully understood.

The accumulation of AGEs in the vessel wall may impair vascular cell structure and function. Furthermore, AGEs may modify the extracellular matrix (ECM) through direct modification of RGD (arg-gly-asp) motifs, causing loss of charge and structural distortion. The ECM has been shown to potentiate VEGF signaling by interacting with cell surface integrins. Vitronectin (VN), one of the components of the ECM, is a multifunctional glycoprotein that is present in the plasma and is localized into the ECM of various tissues. Most plasma VN is an inactive monomer.^10^ ECM VN is present as an active multimeric form that binds to various ligands, such as integrins, plasminogen activator inhibitor-1 and urokinase receptors. VN also has an important role in regulating VEGF-induced angiogenesis. A cooperative binding interaction between VEGF receptor-2 (VEGFR-2) and *α*v*β*3 integrin has a key role in regulating VEGF signaling in endothelial cells.^11^ This receptor cross-talk depends on the binding of *α*v*β*3 to VN.^12, 13^ It has been demonstrated that glycation alters some functional properties of collagen,^14, 15, 16^ laminin,^16, 17, 18^ fibronectin^17, 19, 20^ and VN.^20^ However, no previous studies have implicated the glycation of VN in VEGF signaling.

In this study, we glycated VN *in vitro* using methylglyoxal (MGO) and characterized the structure of glycated and unmodified VN. We further tested the hypothesis that the glycation of VN contributes VEGF-mediated endothelial cell activation by disrupting VEGFR-2–*α*v*β*3 cross-talk.

## Results

### Identification of specific glycation sites in human plasma VN

**Figure 1:**
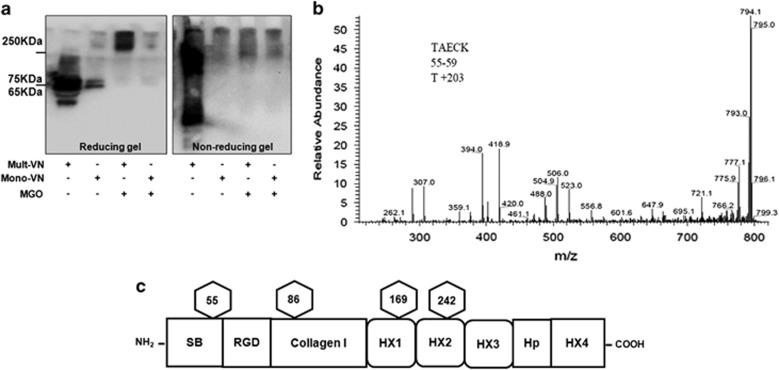
Characterization of glycation of VN by MGO. (**a**) Glycation of VN. Multimeric VN (10 *μ*g/ml) and monomeric VN (10 *μ*g/ml) were treated with MGO (500 *μ*mol/l) at 37 °C for 72 h and samples were separated by SDS-PAGE under reducing and non-reducing conditions. Mult-VN, multimeric VN; Mono-VN, monomeric VN; MGO, methylglyoxal. (**b**) Mass spectra from the glycated VN reveals *O*-glycosylation on T55 in the tryptic peptide TAECK. (**c**) Schematic domain organization of human VN. Glycosylation sites linked to one *O*-glycosylation onT55 and three *N*-glycosylation sites at N86, N169 and N242 are marked by hexagons

To explore whether glycation could be involved in causing VN conformational change, we examined VN expression by SDS-polyacrylamide gel electrophoresis (PAGE) under reducing and non-reducing conditions using 5–20% gradient gels. Multimeric VN or monomeric VN were incubated with MGO for 72 h at 37 °C. Western blotting analysis of incubates was performed using anti-human VN antibody. As shown in Figure 1a, normal VN-positive bands (65/75 kDa) vanished in multimeric VN and monomeric VN in the presence of MGO and multimeric-VN treated by MGO clearly shifted to a higher molecular mass, which indicated the changes in glycosylation and the existence of covalently cross-linked products.^5^ The production of glycated VN (VN-AGEs) was identified with fluorescence spectrophotometer measuring AGE-specific fluorescence at an emission of 440 nm and an excitation of 370 nm. We observed that the fluorescence of VN-AGEs was about three times as much as unmodified VN (Supplementary Figure 1a), suggesting VN-AGEs had been successfully produced *in vitro*.

To unequivocally identify glycated sites and peptides, glycated VN were digested using trypsin and PNGaseF followed by LC–ESI–MS/MS system analysis. The amino acid sequence of VN is displayed, showing glycosylation sites identified in this study, and an *O*-glycan attachment site was found (T44) (Supplementary Figure 1b). In total, 18 peptides were identified by their fragmentation patterns in the glycated VN. These peptides were annotated according to the amino acid sequence and their details are listed in Table 1. The results of peptide mass spectra from the glycated VN revealed *O*-glycosylation on T55 in tryptic peptide TAECK (Figure 1b). The mass of the modification was 203, which suggested that the *O*-glycan is *N*-acetylglucosamine, indicating that VN-AGEs were successfully produced *in vitro*. Furthermore, we observed that the *O*-glycan site overlapped with the somatomedin B domain and it was located near the RGD-containing peptide. In addition, the three *N*-glycosylation sites were identified from the sequence of VN. The results from these analyses are summarized in Figure 1c.

### Glycation of VN impairs VEGFR-2 signaling

**Figure 2:**
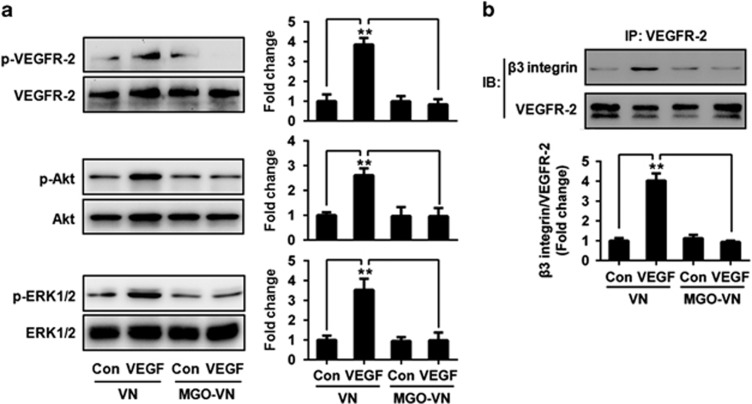
Glycation of VN inhibits VEGF signaling. (**a**) Glycated VN inhibits VEGF-induced VEGFR-2 activation. HUVEC cells were cultured on VN or glycated VN and stimulated by VEGF (50 ng/ml) or vehicle control for 10 min. Cell lysates were prepared and subjected to western blotting, to detect the phosphorylation (p) of VEGFR-2, Akt and ERK1/2, and total VEGFR-2, Akt and ERK1/2. Representative images of three independent experiments and densitometric analysis of phosphorylated VEGFR-2, Akt and ERK1/2 normalized to total VEGFR-2, Akt and ERK1/2 are shown. Data are shown as mean±S.D. for triplicate experiments and presented as fold changes. **P<0.01. (**b**) Glycation of VN inhibits VEGF-induced VEGFR-2–*β*3 integrin complex formation. HUVEC cells were cultured on VN or glycated VN and stimulated by VEGF (50 ng/ml) or vehicle control for 10 min. Cell lysates were prepared and incubated with a resin-bound anti-VEGFR-2 antibody. Captured proteins were analyzed by western blotting with anti-*β*3 integrin antibodies. Representative images of three independent experiments and densitometric analysis are shown. Data are shown as mean±S.D. and are presented as fold changes. **P<0.01

VN is a major ligand for *α*v*β*3 and it significantly enhances VEGF-mediated activation of endothelial cells via VEGFR-2.^13, 21^ We hypothesized that the glycation of VN may impair VEGF-induced activation of VEGFR-2. To test this hypothesis, human umbilical vein endothelial cells (HUVECs) were cultured in wells coated with unmodified or glycated VN and were exposed to VEGF or vehicle control for 10 min. Cell lysates were then prepared, separated by SDS-PAGE and analyzed by immunoblotting. VEGF significantly increased VEGFR-2 phosphorylation in HUVEC cells grown on VN. However, the stimulatory effect of VEGF on VEGFR-2 phosphorylation was inhibited in cells grown on glycated VN (Figure 2a). Intracellular signaling pathways activated by binding of VEGF to VEGFR-2 were also examined. Glycated VN also significantly inhibited VEGF-induced phosphorylation of Akt and extracellular signal-regulated kinase1/2 (ERK1/2) (Figure 2a). As a whole, these results indicated that the glycation of VN impairs VEGFR-2 phosphorylation and downstream signaling in HUVEC cells.

*α*v*β*3 Integrin-augmented VEGFR-2 phosphorylation is dependent on the formation of the VEGFR-2–*α*v*β*3 complex.^11, 12^ Therefore, we examined the effects of glycated VN on the VN-dependent binding interaction between VEGFR-2 and *α*v*β*3. VEGFR-2–*α*v*β*3 integrin complexes were captured by an immobilized anti-VEGFR-2 antibody and detected by immunoblotting with an anti-*β*3 integrin antibody. The results showed that VEGF significantly enhanced the coimmunoprecipitation of VEGFR-2 and *β*3 integrin in HUVEC cells grown on VN, but no enhancement was seen in cells grown on MGO-glycated VN (Figure 2b). These results suggested that VEGF induces the formation of VEGFR-2–*α*v*β*3 integrin complexes, and that the glycation of VN inhibits this process.

### Glycation of VN inhibits VEGF-induced *β*3 integrin phosphorylation

**Figure 3:**
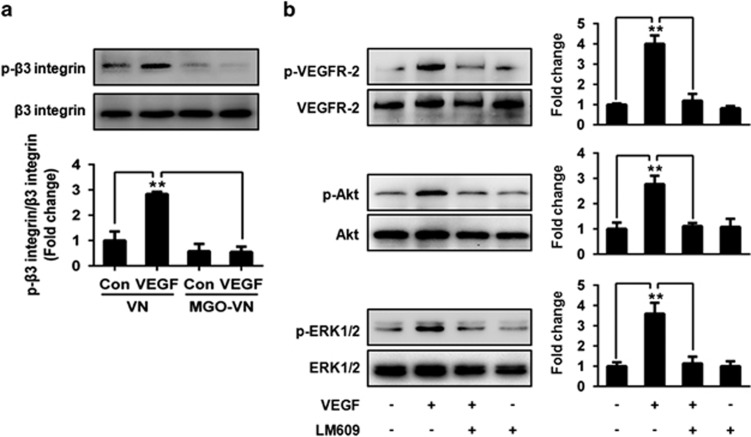
Glycation of VN inhibits VEGF-induced phosphorylation of *β*3 integrin. (**a**) HUVEC cells were cultured on VN or glycated VN and stimulated by VEGF (50 ng/ml) or vehicle control for 10 min. Cell lysates were prepared and incubated with a resin-bound anti-*β*3 integrin antibody. Captured proteins were analyzed by western blotting with anti-phosphotyrosine antibodies. Representative images of three independent experiments and densitometric analysis are shown. Data are shown as mean±S.D. and are presented as fold changes. **P<0.01. (**b**) *α*v*β*3 integrin antibody (LM609) inhibits VEGF-induced VEGFR-2 signaling. HUVEC cells were cultured on VN and were pretreated with LM609 (20 *μ*g/ml) or vehicle control for 30 min, followed by stimulation with VEGF (50 ng/ml) for 10 min. Cell lysates were prepared and subjected to western blotting, to detect the phosphorylation (p) of VEGFR-2, Akt and ERK1/2 and total VEGFR-2, Akt and ERK1/2. Representative images of three independent experiments and densitometric analysis of phosphorylated VEGFR-2, Akt and ERK1/2 normalized to total VEGFR-2, Akt and ERK1/2 are shown. Data are shown as mean±S.D. for triplicate experiments and presented as fold changes. **P<0.01

*β*3 Integrin phosphorylation is complementary to VEGF-induced tyrosine phosphorylation of VEGFR-2. VEGFR-2 activation can induce *β*3 integrin phosphorylation, which in turn is required for VEGFR-2–*α*v*β*3 integrin association and maximum phosphorylation of VEGFR-2.^13^ Therefore, the effects of glycated VN on *β*3 phosphorylation were observed. Stimulation with VEGF for 10 min induced a significant increase in *β*3 phosphorylation in HUVEC cells grown on unmodified VN, but not in those grown on glycated VN (Figure 3a). These results suggested that VN glycation-induced VEGF resistance is associated with an inhibition of *β*3 phosphorylation stimulated by VEGF.

We further examined the effects of blockade of *α*v*β*3 on VEGFR-2 signaling. HUVEC cells grown on VN were pretreated with LM609 or vehicle control for 30 min, followed by stimulation with VEGF for 10 min. Cell lysates were separated by SDS-PAGE and analyzed by immunoblotting. Anti-*α*v*β*3 blocking antibody significantly inhibited VEGF-induced phosphorylation of VEGFR-2, Akt and ERK1/2 (Figure 3b). These results further suggested that *β*3 is required for VEGF-stimulated activation of VEGFR-2.

### Glycation of VN impairs VEGF-induced endothelial cell migration and angiogenesis *ex vivo*

**Figure 4:**
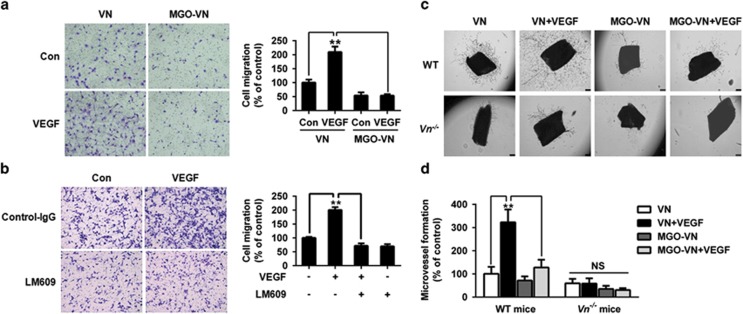
Glycation of VN impairs VEGF-induced endothelial cell migration and angiogenesis *ex vivo*. (**a**) Glycated VN decreases VEGF-induced migration of HUVEC cells. HUVEC cells were added to the upper chambers containing VN or glycated VN-coated porous filters and then incubated with VEGF (50 ng/ml) or vehicle control as shown. After 24 h, cells were fixed and stained with crystal violet. Migrated cells were counted. Representative images of cell migration are shown. Quantitative assessment of triplicate cell migration experiments was performed. Data shown are mean±S.D. and are expressed as % of control (first bar). **P<0.01. (**b**) LM609 inhibits VEGF-induced migration of endothelial cells. LM609 inhibits VEGF-induced migration of HUVEC cells. HUVEC cells were added to the upper chambers containing VN-coated porous filters and were pretreated with LM609 (20 *μ*g/ml) or vehicle control for 30 min. Thereafter, cells were incubated with VEGF (50 ng/ml) or vehicle control as shown. Cells were fixed after 24 h and stained with crystal violet. Migrated cells were counted. Representative images of cell migration are shown. Quantitative assessment of triplicate cell migration experiments was performed. Data shown are mean±S.D. and are expressed as % of control (first bar). **P<0.01. (**c**) Aortic rings from WT and *Vn*^*−/−*^ mice were cultured *ex vivo* for 14 days in Matrigel with VN or glycated VN in the presence of VEGF (50 ng/ml) or vehicle control as indicated. Representative images of microvessel sprouts from the aortic rings are shown. (**d**) Quantitative analysis of microvessel sprouts from the aortic rings. The results are shown as mean±S.D. and are expressed as % of control (first bar). **P<0.01

We examined the effects of glycated VN on the physiological responses of endothelial cells to VEGF stimulation. HUVEC cells were added to the upper chambers containing unmodified or glycated VN-coated porous filters and were exposed to VEGF. After 24 h, cells that migrated to the lower chamber of the membrane were counted. VEGF significantly increased the migration of HUVEC cells grown on unmodified VN. However, the glycation of VN inhibited the migration induced by VEGF (Figure 4a). To further examine the consequences of *α*v*β*3-integrin-blocking antibodies on cell migration, cells were seeded onto VN-coated upper chambers and were pretreated with *α*v*β*3 integrin antibody for 30 min. Cells were then stimulated by VEGF for 24 h. LM609 also significantly inhibited VEGF-induced migration of HUVEC cells (Figure 4b).

To determine the role of glycated VN in VEGF-induced angiogenesis, we cultured segments of the aorta from wild-type (WT) and VN-deficient (*Vn*^−/−^) mice *ex vivo* in Matrigel in the presence or the absence of glycated VN. As shown in Figures 4c and d, VEGF-induced angiogenesis was significantly impaired in *Vn*^*−/−*^mice compared with WT mice. The capacity of VEGF to stimulate microvessel sprouting was significantly greater in unmodified VN than in glycated VN in WT mice. These results suggested that the glycation of VN has a key role in contributing VEGF-induced angiogenesis *in vivo*.

### VN-AGEs formation induces impairment of the angiogenic process in diabetic mice

**Figure 5:**
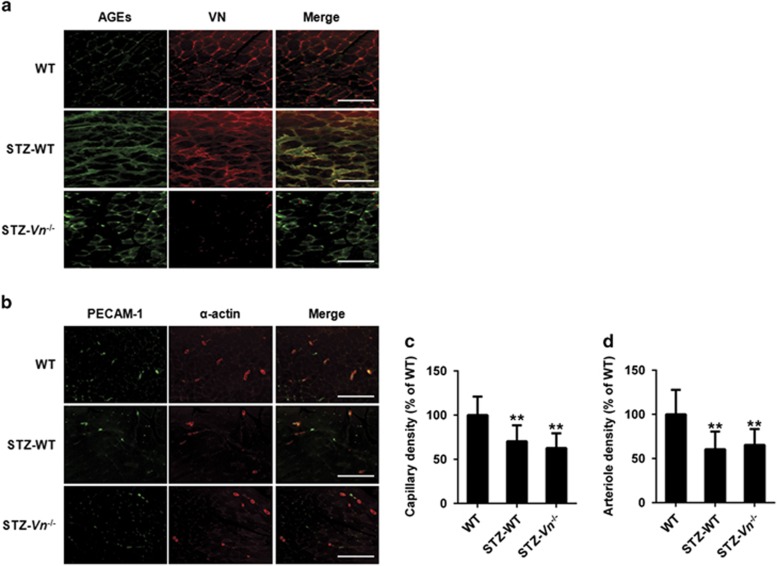
Increased VN-AGEs in ischemic muscles in STZ-induced diabetic mouse hindlimb ischemia model. (**a**) VN was glycated in STZ-induced diabetic mice. Representative images of ischemic gastrocnemius muscle sections from WT, STZ-treated WT mice and STZ-treated *Vn*^*−/−*^mice stained with antibodies directed against AGEs and VN (AGEs, green; VN, red; and colocalization (Merge), yellow). Distance bars, 200 *μ*m. (**b**) Representative images of capillary (assessed by anti-PECAM-1 immunostaining) and arterioles (assessed by anti-smooth muscle *α*-actin immunostaining) in ischemic gastrocnemius muscles recovered 7 days after femoral artery interruption. Distance bars, 200 *μ*m. Mean capillary (**c**) and arteriole density (**d**) in ischemic gastrocnemius muscles were also shown (*n*=3 per group). Data shown are mean±S.D. and are expressed as % of WT. **P<0.01 *vs* WT

To examine the significance of our findings in a pathological model of VN-AGEs relevant to human cardiovascular disease, we used a severe hindlimb ischemia model of streptozotocin (STZ)-induced diabetic mice (STZ-DM), which produced hyperglycemia. Hindlimb ischemia was induced in STZ-DM mice by ligation and excision of the femoral artery. After 7 days, ischemic gastrocnemius muscles were isolated for immunohistochemical analysis. We observed AGEs formation within the ischemic gastrocnemius muscles and found a marked AGEs accumulation in STZ-WT mice compared with WT mice and STZ-*Vn*^−/−^ mice. The increased merge of AGEs and VN staining was shown colocalization in STZ-WT mice (Figure 5a), providing evidences for AGEs cross-linking of VN in the ischemic muscle of diabetic mice. Furthermore, we observed that capillary and arteriole density in ischemic gastrocnemius muscle 7 days after induction of ischemia was significantly higher in WT mice than in STZ-WT mice and STZ-*Vn*^−/−^ mice (Figures 5b–d).

## Discussion

Hyperglycemia and diabetes mellitus have direct effects on the vessel wall by promoting glycation and cross-linking of long-lived ECM, leading to the production of one form of the AGEs, which has been implicated in diabetic vascular complications. Studies suggest that the formation and accumulation of VN have been proposed to be involved in the evolution of diabetic microangiopathy.^3, 4^ However, it is not fully understood what leads to VN accumulation and in which form VN exerts its antiangiogenic effects. In this study, we have investigated the effects of MGO modification of VN on conformational and structural properties. We synthesized glycated VN *in vitro* and evaluated the modification. Our findings were similar to a previous study, which observed that a conformational change in glycated VN yielded high-molecular-weight SDS-resistant products.^5^ By mass spectrometry analysis, we observed that the glycated peptide is overlapped by the plasminogen activator inhibitor-1-binding domain (Somatomedin B domain), which is located near the RGD-containing peptide. AGE formation leads to a reduction in the binding of collagen and heparan sulfate to VN.^6^ The characterized altered structure of glycation on VN results in the loss of binding between RGD-binding integrins and their ligands. Thus, it is likely to be that the alteration blocks the adhesion and migration of endothelial cells, thereby inhibiting angiogenesis.

The interaction between multimeric VN and *α*v*β*3 integrin is known to potentiate VEGF-induced angiogenesis.^11, 12^ The results of the present study showed significantly decreased VEGFR-2 phosphorylation and downstream signaling activation in HUVEC cells grown on MGO-glycated VN compared with cells grown on unmodified VN, suggesting a serious impairment of glycated VN in VEGF signaling. Previous studies showed that *α*v*β*3 integrin and VN potently potentiate VEGFR-2 activation by VEGF,^9, 22^ demonstrating a critical role of *α*v*β*3–VEGFR-2 cross-talk in VEGF signaling and the VN-dependent interaction. Our experiments suggest that the modification of the *α*v*β*3–VN binding interaction by the presence of glycosylation directly uncouples *α*v*β*3–VEGFR-2 cross-talk and downregulates VEGFR-2 activation. Specifically, MGO modification of arginine residues within the RGD and GFOGER motifs can lead to integrin inactivation and disengagement from the ECM.^23^

Numerous reports suggest that VEGF is a critical growth factor for angiogenesis under pathological conditions and EC migration is a key event for angiogenesis *in vivo*. Using a solid-phase assay, we previously showed that multimeric VN binds VEGF, and such interactions could localize VEGF and further bind to VEGFR-2. Our data suggest that the pro-angiogenic (endothelial cell migration and tube sprouting) effect of VN was lost by conformational changes in *α*v*β*3 integrin and/or VEGF-VN binding under glycosylation.

Alternatively, VN is believed to interact with collagen and VN–collagen interactions regulate VN-mediated cell adhesion and migration. We and others observed that collagen binding of VN can be modulated by its glycosylation status.^24, 25^ Thus, the presence of glycosylation on VN decreases its binding activity, which results in an inhibitory effect on cell adhesion and migration, suggesting that the proangiogenic effect of VEGF requires physical binding to VN.

The attenuated VEGF signal transduction, or VEGF resistance, has been established as one of the mechanisms underlying the dysfunction of angiogenesis in people with type 2 diabetes.^2, 9^ Our experiments involving cell migration *in vitro* and microvessel sprouting from aortic rings *ex vivo* demonstrated that glycated VN significantly decreased VEGF-induced cell migration and vessel outgrowth. Consistent with these findings, we showed evidences for AGEs cross-linking of VN in the ischemic muscle of diabetic mice, and associated with an impairment in capillary and arteriole density after induction of hindlimb ischemia. Previous studies have shown that collateral arteriole development after femoral artery occlusion are dependent on VEGFR-2 activation by VEGF.^26, 27^ Therefore, our results support the *in vivo* relevance of the inhibition of VEGFR-2 activation by the formation of VN-AGEs. A limitation of our hindlimb ischemia model experiment is that we cannot definitively conclude the negative effect of VN-AGE formation on VEGFR-2 activation, as formation of VN-AGEs could potentially modulate the angiogenic response to ischemia by VEGFR-2-independent pathways. Nevertheless, our hindlimb ischemia model data support the significance of our proposed molecular mechanisms in a clinically relevant *in vivo* context, thereby complementing our cell culture, and *ex vivo* aortic ring data, which demonstrated that glycated VN inhibits VEGFR-2 activation. Additional *in vivo* studies will be necessary to further dissect and better characterize the significance of our newly reported regulatory pathway on VEGF-dependent angiogenic signaling in other disease models and diabetic patient samples.

In summary, we have found that the formation of glycated VN by MGO inhibits the pro-angiogenic effect of VEGF with mechanisms involving the inactivation of the VEGFR-2 pathway and disruption of the pro-angiogenic binding interaction between VEGFR-2 and *α*v*β*3. These data reveal that the underlying mechanism of diabetic microangiopathy may be through increased formation of glycated VN.

## Materials and Methods

### Reagents and chemicals

MGO, STZ and human VN were from Sigma (St Louis, MO, USA). Recombinant VEGF-A was from R&D Systems (Minneapolis, MN, USA). Growth-factor-reduced BD Matrigel Matrix (BD Biosciences, San Jose, CA, USA) was from Chemicon International (Temecula, CA, USA). Antibodies to phosphorylated VEGFR-2, total VEGFR-2, phosphorylated Akt, total Akt, phosphorylated ERK1/2, total ERK1/2 and *β*3 integrin were form Cell Signaling Technology (Beverly, MA, USA). Antibodies to platelet endothelial cell adhesion molecule-1 (PECAM-1) and smooth muscle *α*-actin were form Santa Cruz Biotechnology (Santa Cruz, CA, USA). Antibody to AGEs was from Abcam (Cambridge, MA, USA). Antibody to VN was from R&D Systems. LM609 (anti-*α*v*β*3 antibody) and anti-phosphotyrosine antibodies were from Merck Millipore (Watford, UK).

### Cell culture

HUVEC cells (Cascade Biologics, Portland, OR, USA) were grown in Medium 200 (Cascade Biologics) containing low-serum growth supplement. Cells used were passaged 3–7 times.

### Animals

C57BL/6J mice were from Jackson Labs (Bar Harbor, ME, USA). C57BL/6J-congenic *Vn*^*−/−*^ mice were a gift from Dr David Ginsburg, University of Michigan, Ann Arbor, MI, USA.^28^ All animal care and experimental procedures were approved by the University of Missouri Animal Care and Use Committee.

### Glycation of VN

Glycation of VN was performed as described previously.^17^ Briefly, VN was modified by incubating the protein (10 *μ*g/ml) with MGO (500 *μ*M) in 100 mM sodium phosphate buffer, pH 7.4, at 37 °C for 72 h. Control VN was subjected to the same conditions, except that MGO was omitted. The production of VN-AGEs was identified with western blotting and fluorescence spectrophotometer (Molecular Devices, Sunnyvale, CA, USA) measuring AGE-specific fluorescence at emission of 440 nm and excitation of 370 nm.

Endopeptidase trypsin (modified, sequencing grade) was purchased from Promega (Madison, WI, USA). Pepcin was obtained from Roche (Indianapolis, IN, USA). PNGase F was purchased from New England Biolabs (Ipswich, MA, USA). All other chemicals and enzymes used in proteolytic digestion and high-performance liquid chromatography (HPLC) were obtained from Sigma. The quadrupole ion trap mass spectrometer (LTQ) used in the proteomic analysis was manufactured by Thermo (Palo Alto, CA, USA). The VN sample was denatured in 8 M urea, treated with DTT to reduce disulfide bonds and then reacted with iodoacetamide to alkylate cysteine residues. The sample was cleaned up by dialysis to remove all chemicals. The cleaned sample was digested with trypsin and then treated with PNGase F.

The digested peptide mixture was analyzed by an LC–ESI–MS/MS system, in HPLC for which a 75-*μ*m inner diameter reverse-phase C18 column was on-line coupled with an ion trap mass spectrometer. The solvents used for HPLC were solvent A (98% H_2_O, 2% acetonitrile) and solvent B (10% H_2_O, 90% acetonitrile), both containing 0.025% TFA. The analysis time was 200 min for solution-digested samples. The data analysis was carried out with ProtMody software suites (ProtTech Inc., Phoenixville, PA, USA). All MS/MS data were searched by SV Finder software (Ver 1.2, ProtTech Inc.), to look for potential sequence modification. All hits from the search were manually validated and confirmed by manual MS/MS spectral assignment.

### Cell migration assays

HUVEC cell migration assays were performed using transwell migration chambers with a 8.0-*μ*m-size porous membrane (Corning Costar, Corning, NY, USA). The membranes were precoated with VN or MGO-glycated VN overnight at 4 °C, after which HUVEC cells (2 × 10^4^) were added to the upper chambers and treated with VEGF (50 ng/ml) or vehicle control. In some experiments, cells were seeded onto VN-coated wells and pretreated with *α*v*β*3 integrin antibody, LM609 (20 *μ*g/ml) for 30 min,^29^ followed by stimulation with VEGF (50 ng/ml). After 24 h incubation at 37 °C in a humidified chamber with 5% CO_2_, porous membranes were rinsed and cells remaining in the upper chamber were removed by a cotton swab. Membranes were fixed and then stained with 0.5% crystal violet. Cells that migrated to the lower chamber were counted.

### Immunoblotting

HUVEC cell lysates were prepared as described.^30^ Supernatants were separated by SDS-PAGE and transferred onto polyvinylidene fluoride (PVDF) membranes (Bio-Rad Laboratories, Hercules, CA, USA). The membranes were blocked with 5% nonfat dry milk solution for 1 h at room temperature. For phosphorylation of VEGFR-2, Akt and ERK1/2, the membranes were incubated with rabbit or mouse IgG raised against phosphorylated VEGFR-2 (1 : 500), Akt (1 : 1000) and ERK1/2 (1 : 1000), respectively. For total VEGFR-2, Akt and ERK1/2, anti-VEGFR-2 (1 : 500), anti-Akt (1 : 1000) and anti-ERK1/2 (1 : 1000) antibodies, respectively, were used on the same blots after stripping. Secondary antibody was horseradish-peroxidase (HRP)-conjugated goat IgG raised against rabbit or mouse IgG (Santa Cruz Biotechnology). After further washes, blots were developed with ECL substrate (Merck Millipore).

To measure *β*3 integrin phosphorylation, cell lysates were incubated with anti-*β*3 integrin antibody overnight at 4 °C, with gentle rotation. Protein A/G Plus-agarose beads were added to each sample and samples were incubated at 4 °C for additional 4–6 h. Immune complexes were captured, solubilized by 50 *μ*l sample buffer, separated by SDS-PAGE and transferred to PVDF membrane. The membrane was incubated with anti-phosphotyrosine antibody or anti-*β*3 integrin antibody. Secondary antibody was HRP-conjugated goat IgG raised against rabbit or mouse IgG (Santa Cruz Biotechnology). After further washes, blots were developed with ECL substrate (Merck Millipore).

### Immunoprecipitation

HUVEC cells were cultured on wells precoated with VN or MGO-VN and stimulated by VEGF (50 ng/ml) for 10 min. Protein immunoprecipitation was performed with IP kit (Pierce, Rockford, IL, USA). Cells were collected with lysis buffer and clarified by 12 000 × *g* for 20 min. Equal amounts of protein were incubated with anti-VEGFR-2 antibody overnight at 4 °C, with gentle rotation. Protein A/G Plus agarose beads were added to each sample and samples were incubated at 4 °C for additional 4–6 h. Immune complexes were captured according to the instructions of the IP kit and solubilized by the addition of 50 *μ*l of SDS sample buffer. Next, immune complexes were analyzed by immunoblotting. VEGFR-2 and *β*3 integrin subunit were detected with appreciate antibodies. Secondary antibody was HRP-conjugated goat IgG raised against rabbit IgG (Santa Cruz Biotechnology).

### *Ex vivo* tissue culture

Tissue culture was performed as described previously, with minor modifications.^31^ Briefly, thoracic aortic rings isolated from WT and *Vn*^*−/−*^ mice were embedded between two layers of growth-factor-reduced Matrigel (250 *μ*l/layer) in a 24-well plate, in the presence of VN or glycated VN, followed by exposing to Medium 200 (Cascade Biologics; 500 *μ*l) with or without VEGF (50 ng/ml). Medium was changed every 3 days. Aortic rings were photographed 14 days later. The number of sprouting microvessels was quantified by computer-assisted images analysis using Image-Pro Plus software.^32^

### Mouse diabetes model

To induce diabetes, mice were injected i.p. with 55 mg/kg STZ in 0.05 M sodium citrate buffer, pH 4.5, daily for 5 days. Seven days after the last injection, blood glucose levels were measured. Only mice with blood glucose levels ≥11.1 mM were used in the present study. Age-matched non-diabetic mice were injected with sodium citrate buffer and served as controls. After 3 weeks of treatment, mouse hindlimb ischemia model was performed in untreated control and STZ-induced diabetic mice.

### Mouse hindlimb ischemia model

Unilateral hindlimb ischemia was induced in mice by ligation and excision of a segment of the left femoral artery, as previously described.^31^ Mice were euthanized 7 days after surgery. Ischemic gastrocnemius muscle was excised, embedded in paraffin and cross-sections were prepared for immunohistochemical analysis.

### Immunohistochemistry

Tissue sections were incubated with rabbit antibody directed against AGE formation (10 *μ*g/ml) and FITC-conjugated species-specific secondary antibody (1 : 200). Costaining was then performed with a rat antibody directed against VN (20 *μ*g/ml) and Texas red-conjugated species-specific secondary antibody (1 : 200). Arterioles within gastrocnemius muscle were immunostained with anti-smooth muscle *α*-actin antibody (1 : 100) and capillaries within gastrocnemius muscle were immunostained with anti-PECAM-1 antibody (1 : 100).

### Data analysis

Data are presented as mean± S.D. of mean. Results were analyzed by one-way analysis of variance followed by *post hoc* comparison. The level of significance was set at *P*<0.05.

## Figures and Tables

**Table 1:** VN peptides detected by LC–MS/MS

**Scan**	**Peptide**	**Start**	**Stop**	**Calculated mass**	**Measured mass**
4613	CTEGFNVDKK	28	37	1196.5	1196.9
4168	TAECK T+203	55	59	810.3	810.8
7630	GNPEQTPVLKPEEEAPAPEVG	108	128	2187.1	2186.3
5860	PFDAFTDLK	160	168	1052.5	1052.0
7296	AFRGQYCYELDEK	174	186	1677.7	1677.4
3378	AVRPGYPK	187	194	886.5	886.3
11 410	LIRDVWGIEGPIDAAFTR	195	212	2028.1	2029.4
11 294	DVWGIEGPIDAAFTR	198	212	1645.8	1645.1
4870	TYLFK	219	223	670.4	670.0
3738	GSQYWR	224	229	795.4	795.1
7840	FEDGVLDPDYPR	230	241	1421.6	1421.2
2370	TSAGTR	325	330	591.3	590.3
6629	HGVPGQVDAAMAGR	340	353	1364.7	1364.3
3916	PSLAK	363	367	514.3	514.4
3206	FFSGDK	438	443	699.3	699.8
5068	YYRVNLR	444	450	982.5	981.9
5702	VDPPYPR	457	463	842.4	842.5
10 263	SIAQYWLGCPAPGHL	464	478	1668.8	1668.2

Abbreviations: LC, liquid chromatography; MS/MS, tandem mass spectrometry; VN, vitronectin

## Abbreviations

AGE: advanced glycation end product
ECM: extracellular matrix
ERK: extracellular signal-regulated kinase
HUVEC: human umbilical vein endothelial cell
MGO: methylglyoxal
VEGF: vascular endothelial growth factor
VEGFR-2: VEGF receptor-2
VN: vitronectin
PECAM-1: platelet endothelial cell adhesion molecule-1
STZ: streptozotocin
