# Supplementary material for: Glycation of vitronectin inhibits VEGF-induced angiogenesis by uncoupling VEGF receptor-2–αvβ3 integrin cross-talk
Source: Cell Death Dis. 2015 Jun 25;6(6):e1796–. doi: 10.1038/cddis.2015.174 (PMC4669844; doi:10.1038/cddis.2015.174)
Supplement: Supplementary Information [file cddis2015174x1.pdf]

Supplementary Figures

Supplementary Figure 1 Characterization of glycation of VN by MGO. (**a**) Identification of glycated VN. VN (10 mg/ml) was incubated with MGO (500 μM) in 100 mM sodium phosphate buffer, pH 7.4, at 37 °C for 72 h. Control VN was subjected to the same conditions except that MGO was omitted. The fluorescence intensity was measured at 370/440 nm in the fractions. Results represent the mean ± SEM for triplicate determinations. **P*<0.05 vs. unmodified VN. (**b**) Amino acid sequences of human VN (478 AA). Glycosylation sites were identified in this study. The three N-glycosylation sites were colored in blue (rectangle). A *O*-glycan attachment site was colored in yellow (T55, hexagon). Covered peptides were shaded in red.

a.

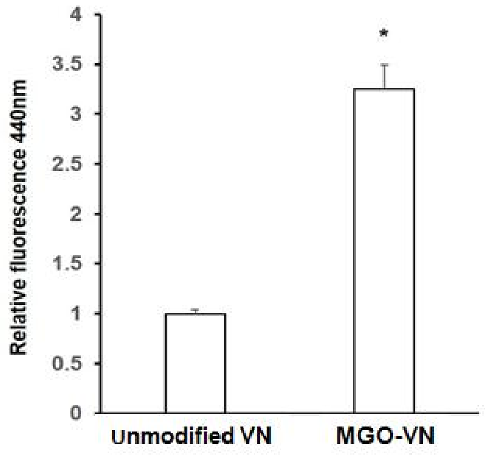

b.

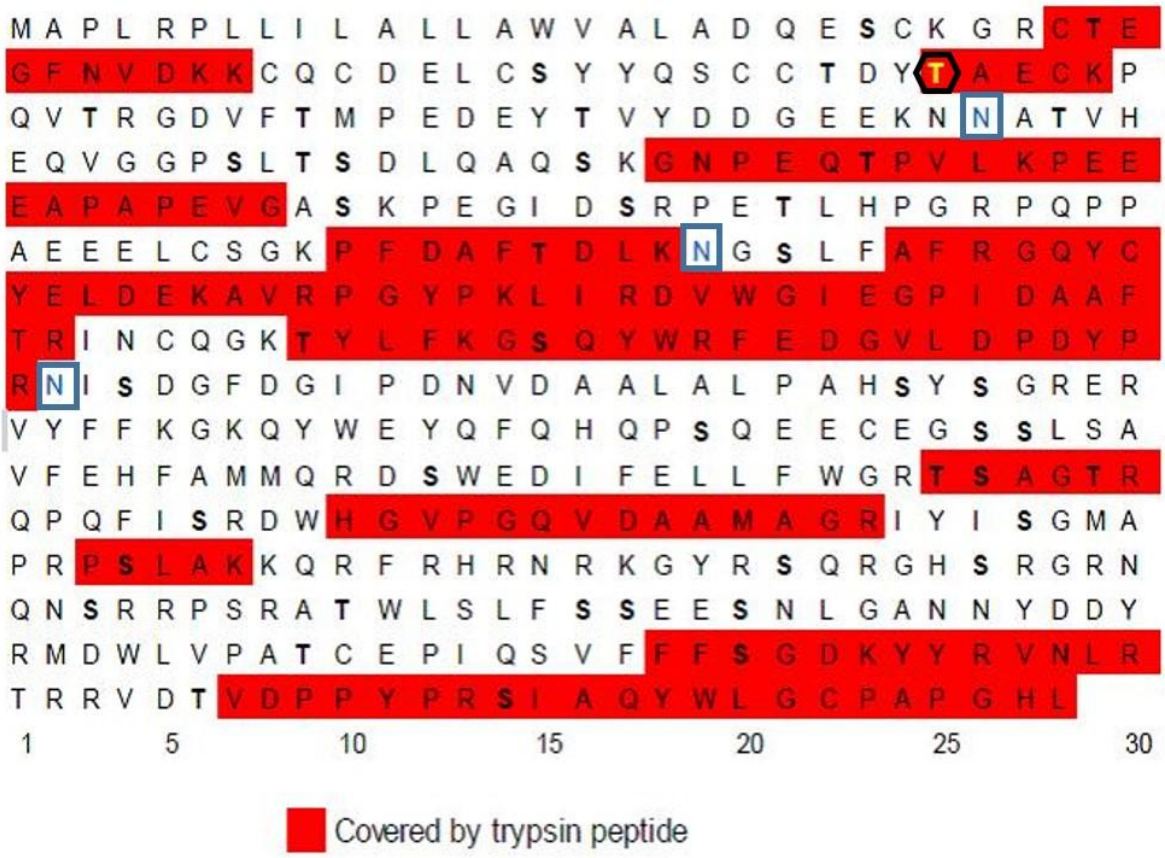
MAPLRPLLILALLAWVALADQESCKGRCTE
GFNVDKKCQCDELCSYYQSCCTDYTAECKP
QVTRGDVFTMPEDEYTVYDDGEEKNNATVH
EQVGGPSLTSDLQAQSKGNPEQTPVLKPEE
EAPAPEVGASKPEGIDSRPETLHPGRPQPP
AEEELCSGKPFDAFTDLKNGSLFAFRGQYC
YELDEKAVRPGYPKLIRDVWGIEGPIDAAF
TRINCQGKTYLFKGSQYWRFEDGVLDPDYP
RNISDGFDGIPDNVDAALALPAHSYSGRER
VYFFKGKQYWEYQFQHQPSQEECEGSSLSA
VFEHFAMMQRDSWEDIFELLFWGRTSAGTR
QPQFISRDWHGVPGQVDAAMAGRIYISGMA
PRPSLAKKQRFRHRNRKGYRSQRGHSRGRN
QNSRRPSRATWLSLFSSEESNLGANNYDDY
RMDWLVPATCEPIQSVFFFSGDKYYRVNLR
TRRVDTVDPPYPRSIAQYWLGCPAPGHL
1 5 10 15 20 25 30
Covered by trypsin peptide
